# Simuliids (Diptera: Simuliidae) from Eastern Andalusia (Spain): Update and New Contributions

**DOI:** 10.3390/insects17030267

**Published:** 2026-03-02

**Authors:** David López-Peña, Matúš Kúdela, Tatiana Kúdelová, Antonio Ricarte, José Vicente Falcó-Garí

**Affiliations:** 1 Laboratory of Entomology and Pest Control, Institut Cavanilles de Biodiversitat i Biologia Evolutiva (ICBiBE), Universitat de València (Estudi General), C/Catedrático José Beltrán, 2, 46980 Paterna, Spain; j.vicente.falco@uv.es; 2 Departamento de Ciencias del Mar y Biología Aplicada, Campus (Zonas Comunes)—PB—(0000PB997), Universidad de Alicante, Ap. de Correos, 99, 03080 Alicante, Spain; 3 Department of Zoology, Faculty of Natural Sciences, Comenius University, Ilkovičova 6, SK-84215 Bratislava, Slovakia; matus.kudela@uniba.sk (M.K.); tatiana.kudelova@uniba.sk (T.K.); 4 Research Institute CIBIO (Centro Iberoamericano de la Biodiversidad), Science Park, University of Alicante, Ctra. San Vicente del Raspeig s/n, 03690 San Vicente del Raspeig, Spain; antonio.ricarte@ua.es

**Keywords:** blackflies, new records, species checklist, distribution, altitudinal range, public health, veterinarian importance

## Abstract

This study enhances and improves the current knowledge of blackfly diversity in the mountainous regions of Eastern Andalusia, with a focus on how simuliid species are distributed through altitude. It provides an updated checklist of species recorded in the provinces of Almería, Granada, and Jaén, by integrating published data with new records from the field. This study identified 16 blackfly species based on morphological criteria, increasing the number of known simuliid species in the region from 25 to 28. Three species—*Simulium brevidens* (Rubtsov, 1956), *Simulium quasidecolletum* Crosskey, 1988, and *Simulium trifasciatum* Curtis, 1839—are reported in Granada for the first time. Most of the species were recorded within their previously known altitudinal range; however, *Simulium equinum* (Linnaeus, 1758) and *Simulium lineatum* (Meigen, 1804) were found at higher elevations, and *Simulium urbanum* (Davies, 1966), at lower elevations, resulting in extensions of their known altitudinal ranges. The newly recorded species *S. quasidecolletum* had a narrow altitudinal range (419 m); *S. brevidens* and *S. trifasciatum* were recorded only at one site. The study offers a comprehensive and updated picture of species richness and altitudinal ecology of blackflies in South-eastern Spain.

## Simple Summary

Blackflies (Diptera: Simuliidae) represent an ecologically and medically significant family of hematophagous insects. The present study aims to enhance understanding of blackfly biodiversity in the mountainous regions of Almería, Granada, and Jaén, South-eastern Spain, with particular emphasis on species diversity and altitudinal distribution patterns. Researchers examined and sampled 24 stream sites and identified blackfly larvae and pupae using morphological features. Consequently, the list of species known from the region has been updated, registering 16 species in this study and discovering three species not previously recorded for Granada, —*Simulium brevidens* Rubtsov, 1956, *Simulium quasidecolletum* Crosskey, 1988, and *Simulium trifasciatum* Curtis, 1839, —thus increasing the total number of known species for the three provinces from 25 to 28. The majority of blackfly species were recorded within their previously known altitudinal range, but in the case of the species *Simulium equinum* (Linnaeus, 1758), *Simulium lineatum* (Meigen, 1804), and *Simulium urbanum* Davies, 1966, their altitudinal ranges were extended. Among the newly recorded species, *S. quasidecolletum* had a narrow altitudinal range (419 m), whereas *S. brevidens* and *S. trifasciatum* were recorded only at one site. The studied mountains host a richer and more diverse blackfly community than previously recognised, and further research is desirable.

## 1. Introduction

Blackflies are known mainly as hematophagous insects, as females of the vast majority of blackfly species feed on the blood of vertebrates. Although only a small proportion of blackfly species are known to transmit parasitic diseases, they are responsible for the transmission of human onchocerciasis (river blindness), which causes severe itching, disfiguring skin lesions, and visual impairment [1]. In addition, blackflies transmit animal onchocerciasis and avian leucocytozoonosis, diseases that can result in substantial economic losses in animal production. Therefore, blackflies are among the most important vectors of parasitic diseases [2]. However, even if blackflies do not transmit pathogens, they are capable of causing serious health and economic damage through their attacks and bites. For example, in livestock, there are known cases of animal deaths, changes in behaviour, or reductions in meat or milk production [2].

In contrast, blackfly larvae play a key role in aquatic ecosystems as filter feeders. By ingesting suspended organic and inorganic particles from the water column, the larvae metabolise this material and excrete it as faecal pellets, thereby transforming fine particulate matter into bioavailable resources for other invertebrate and vertebrate organisms [3].

Therefore, a thorough understanding of blackfly diversity and distribution is essential for advancing research and for effective public health and pest management strategies. Recently, Spain has been one of the European countries where problems with blackflies attacking humans and livestock occur relatively frequently. The high-altitude mountain ranges of Andalusia, such as the Sierra de Los Filabres, Sierra Nevada, and Sierra de Cazorla and Segura, located in the provinces of Almería, Granada, and Jaén, respectively, provided refuge for various species typical of mountain habitats and have allowed their presence until today. The interest of this area is due to its great diversity of habitats and altitudinal breadth that could have acted as a local glacial refuge during the last Ice Age, which took place 20,000 years ago [4,5].

The study of blackflies (Diptera: Simuliidae) in Andalusia, Southern Spain, has been conducted by several national and external worldwide experts throughout history [6,7,8,9,10,11,12,13,14,15]. Their outstanding performances have contributed significantly to both the knowledge of the species diversity of this group of dipterans and their geographical distribution in this region of the world. For all these reasons, and to contribute to the knowledge of blackflies from Spain, the main aim of the present study has been to delve into the species diversity of simuliids from the mountainous regions of the aforementioned provinces and obtain insights about their distribution and altitudinal ranges, emphasising the detection of blood-feeding species and their characteristic geographical distributions.

## 2. Materials and Methods

### 2.1. Area of Study and Sampling Stations

**Figure 1:**
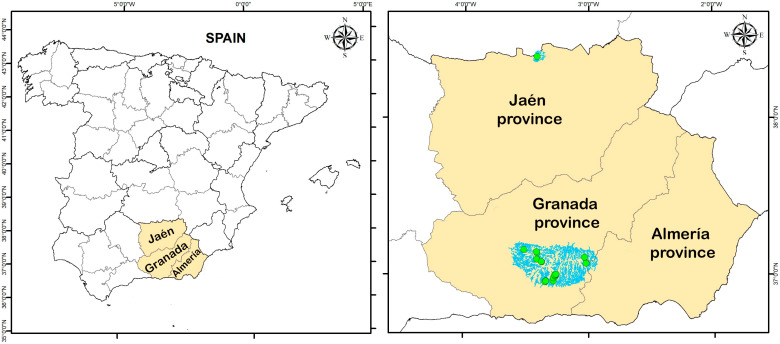
Geographical positioning of the provinces of Almería, Granada and Jaén within Spain (**left side**). Location of the lotic water bodies prospected together with the sampling stations (**right side**). Detailed representation of the water flows, and each sampling station is available in the Supplementary Material (Figures S1–S4).

**Figure 2:**
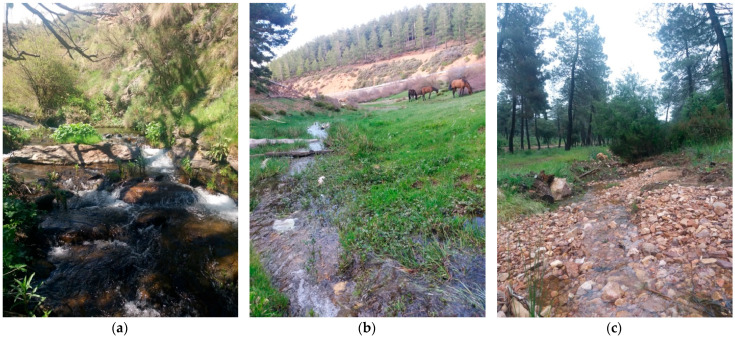
Photographs of several blackfly breeding sites of the study area in South-eastern Spain: (**a**) Stream del Palancón (Almería), (**b**) Stream Hondo (Granada), and (**c**) Stream del Chortal (Jaén). (Photographs: Matúš Kúdela).

During May 2015 and 2018, 24 sampling stations from South-eastern Spain were studied (Figure 1). From these sampling points, two were located in the province of Almería, 21 in Granada province, and one in Jaén province. Data regarding the prospective breeding sites are depicted in Table 1, and the diversity of sampled habitats are shown in Figure 2.

### 2.2. Sample Collection

The potential blackfly breeding sites were thoroughly examined in search of aquatic stages of these dipterans. With this purpose, all types of substrates from lotic water bodies, on which blackfly larvae and pupae tend to adhere, were thoroughly reviewed. Pebbles, stones, rocks, mosses, macrophytes, stalks and leaves of helophytes, bushes and trees in direct contact with the flow of water, as well as fallen tree branches, leaves, or plastic and metal waste, were revised. When detected, larvae and pupae were carefully detached from them with tweezers. Pupae and some larvae were stored in 96.3% of ethanol, while the other part of larvae, especially early younger individuals of the last instar, were stored in modified Carnoy’s solution (ethanol and acetic acid, 3:1); this solution was replaced a minimum of three times subsequently to maintain the purity of the solution.

### 2.3. Sample Processing and Specimen Identification

Once in the laboratory, the collected samples were stored in a refrigerator at a temperature of −22 °C. The processing of the specimens included cleaning the useful identification structures of mud, sand, and other substances that made the morphologically-based identification of larvae and pupae difficult. For more information about the employed sampling and identification methodology, as well as the stereoscopic microscopes used, see [6] (pp. 16,18). The larvae and pupae of blackflies were identified following several identification keys [7,8,9,10,11]. Afterwards, the identified specimens were stored again in the freezer at the Department of Zoology, Faculty of Natural Sciences of Comenius University, Bratislava.

### 2.4. Figure Design and Creation

Photographs of larvae and pupae were taken with a ZEISS Axio Zoom.V16 microscope (Carl Zeiss AG, Oberkochen, Germany) and a Canon EOS 5D camera (Canon, Tokyo, Japan). The images were focus-stacked using Zerene Stacker 1.04. The Geographic Information System (GIS) software ArcMapTM 10.5 of ESRI’s ArcGIS^®^ (Redlands, CA, USA) [12] has been employed to design and generate both the location figure of the study area (Figure 1 and Figures S1–S4) and the updated provincial distribution maps of the species reported for the first time in the researched region.

## 3. Results

### 3.1. Identified Species from the Study Area

**Figure 3:**
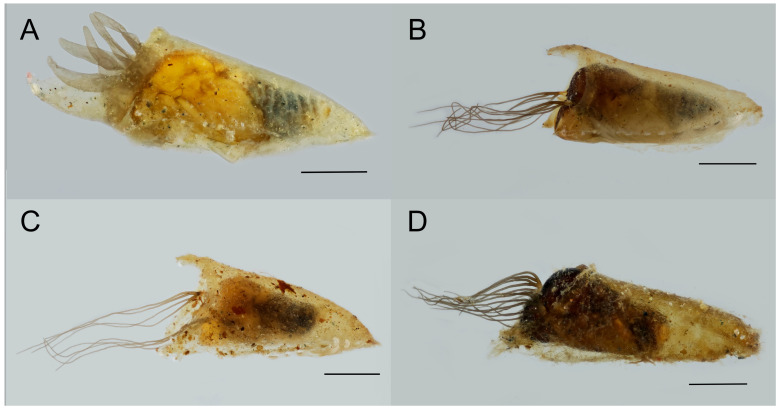
Morphological features of the pupae of various species from the left view: (**A**) *S. equinum*, (**B**) *S. carthusiense*, (**C**) *S. cryophylum*, and (**D**) *S. ornatum* s.l. Scale bar: 1 mm.

**Figure 4:**
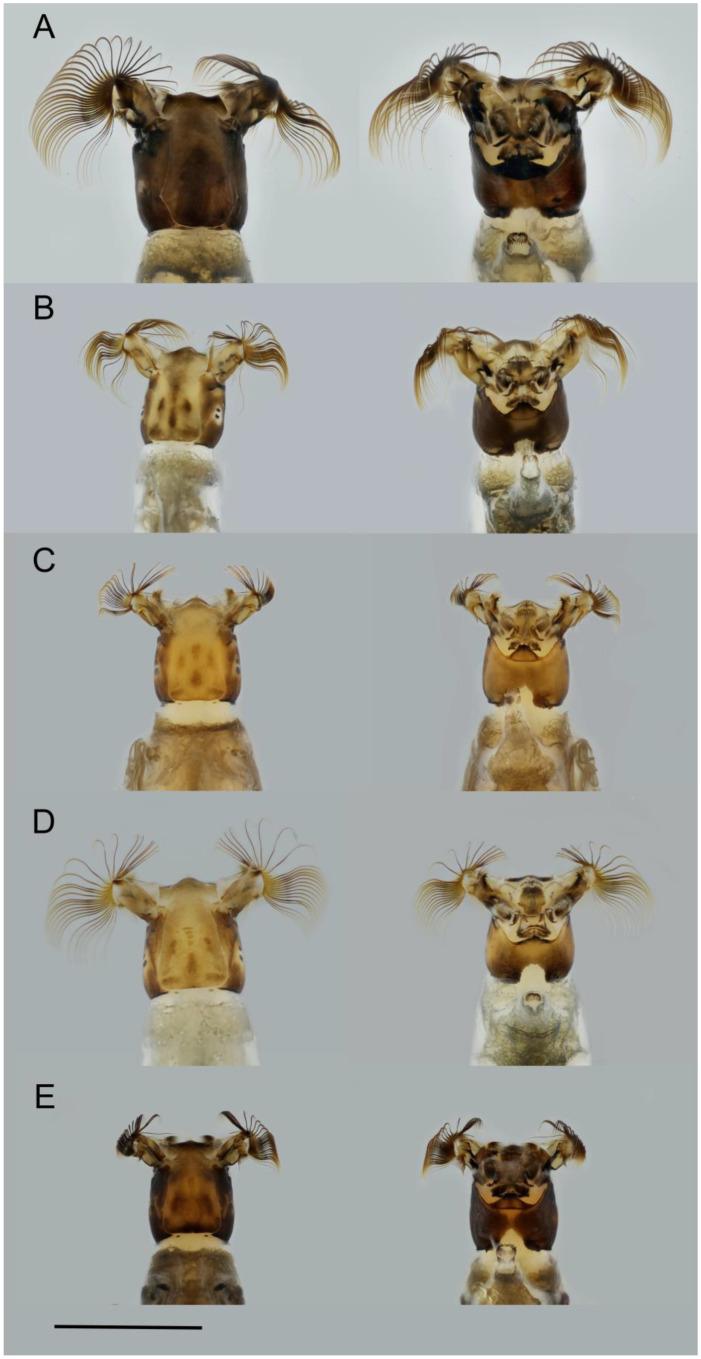
Morphological characteristics of the heads of larvae of several species from the dorsal and ventral views: (**A**) *P. latimucro*, (**B**) *S. petricolum*, (**C**) *S. cryophylum*, (**D**) *S. brevidens*, and (**E**) *S.* sp. aff. *maximum*. Scale bar: 1 mm.

A total of 2731 specimens belonging to 15 nominal and one formally unnamed species (Table 2), four species-groups (*equinum*, *ornatum*, *variegatum* and *vernum*), three subgenera (*Eusimulium* Roubaud, 1906; *Nevermannia* Enderlein, 1921; *Simulium* Latreille, 1802), and two genera (*Prosimulium* Roubaud, 1906 and *Simulium* Latreille, 1802) were collected from 24 sampling sites of the study area, of which 1917 are immature larvae, 508 mature larvae of 15 species, and 306 pupae of nine species. As for immature larvae, if some could be clearly identified to a species level, they are included further in the overview of identified species. Most of the immature larvae, however, could be identified only as belonging to the level of subgenus. Five of the identified species are considered species complexes; they are indicated in Table 2 as s.l. Morphological features of some of the identified species are shown in Figure 3 (pupae) and Figure 4 (larvae).

The most abundant species have been *S. variegatum* and *S. cryophilum* with 1014 and 232 specimens recorded, respectively, followed by *P. latimucro* with 135 individuals, and by *S. equinum* with 42 specimens. By provinces, Granada has reported the highest number of species since the majority of the sample stations are distributed there. The 16 species have been collected from the lotic waters of this region; among them, three are first records from the province of Granada: *S. brevidens*, *S. quasidecolletum*, and *S. trifasciatum*. Likewise, from the province of Jaén, three species have been reported: *S. ornatum*, *S. petricolum*, and *S. rubzovianum*, with *S. petricolum* being the most abundant species (19 specimens). Finally, one species, —*S. cryophilum*, —has been reported from the province of Almería.

An overview of the recorded species and the abundance of their larvae and pupae is summarised in Table 2.

### 3.2. Taxonomic Classification of Simuliids from the Provinces of Almería, Granada and Jaén

The relevance of this study lies in its contribution of three previously undocumented records across the provinces of Almería, Granada, and Jaén: *S. brevidens*, *S. quasidecolletum*, and *S. trifasciatum*. Consequently, the understanding of Simuliidae within this Spanish region has been enriched, offering updated insights into its taxonomic classification, composed of the presence of 28 species. It is noteworthy that, for each species, the capitalised letters (A, G, and J) denote the provinces mentioned in the referenced literature, while (A’, G’, and J’) indicate the provinces from which the species were collected in this study. The updated blackfly species checklist is as follows:

Order Diptera Linnaeus, 1758
  Infraorder Culicomorpha Hennig, 1948
    Superfamily Simulioidea Newman, 1834
      Family Simuliidae Newman, 1834
        Subfamily Parasimuliinae Smart, 1945
          Genus *Prosimulium* Roubaud, 1906
            *hirtipes* species-group
              *Prosimulium latimucro* (Enderlein, 1925)—A, G, G’, J
            Genus *Metacnephia* Crosskey, 1969
            *Metacnephia blanci* (Grenier & Theodorides, 1953)—J
            *Metacnephia nuragica* Rivosecchi, Raastad & Contini, 1975—A
            Genus *Simulium* Latreille, 1802
            Subgenus *Eusimulium* Roubaud, 1906
            *Simulium* (*Eusimulium*) *mellah* Giudicelli & Bouzidi, 2000—A
            *Simulium* (*Eusimulium*) *petricolum* (Rivosecchi, 1963)—A, G, G’, J, J’
            *Simulium* (*Eusimulium*) *rubzovianum* (Sherban, 1961)—A, G, G’, J, J’
            Subgenus *Nevermannia* Enderlein, 1921
            *ruficorne* species-group
              *Simulium* (*Nevermannia*) *angustitarse* (Lundström, 1911)—G, J
              *Simulium* (*Nevermannia*) *ruficorne* Macquart, 1838—A, J
              *vernum* species-group
              *Simulium* (*Nevermannia*) *armoricanum* Doby & David, 1961—G
              *Simulium* (*Nevermannia*) *brevidens* (Rubtsov, 1956)—G’
              *Simulium* (*Nevermannia*) *carthusiense* Grenier & Dorier, 1959—G, G’
              *Simulium* (*Nevermannia*) *cryophilum* (Rubtsov, 1959)—A, A’, G, G’, J
              *Simulium* (*Nevermannia*) *quasidecolletum* Crosskey, 1988—G’
              *Simulium* (*Nevermannia*) *urbanum* Davies, 1966—G, G’
              *Simulium* (*Nevermannia*) *vernum* Macquart, 1826—A, J
            Subgenus *Simulium* Latreille, 1802
            *bezzii* species-group
              *Simulium* (*Simulium*) *bezzi* (Corti, 1914)—A, G, J
              *ornatum* species-group
              *Simulium* (*Simulium*) *intermedium* Roubaud, 1906—A, G, G’, J
              *Simulium* (*Simulium*) *ornatum* Meigen, 1818—G, G’, J, J’
              *Simulium* (*Simulium*) *trifasciatum* Curtis, 1839—G’
              *variegatum* species-group
              *Simulium* (*Simulium*) *argyreatum* Meigen, 1838—G, J
              *Simulium* (*Simulium*) sp. aff. *maximum*—G’
              *Simulium* (*Simulium*) *variegatum* Meigen, 1818—A, G, G’, J
              *Simulium* (*Simulium*) *xanthinum* Edwards, 1933—J
            Subgenus *Trichodagmia* Enderlein, 1934
            *albellum* species-group
              *Simulium* (*Trichodagmia*) *galloprovinciale* Giudicelli, 1963—J
            Subgenus *Wilhelmia* Enderlein, 1921
            *equinum* species-group
              *Simulium* (*Wilhelmia*) *equinum* (Linnaeus, 1758)—G, G’, J
              *Simulium* (*Wilhelmia*) *lineatum* (Meigen, 1804)—G, G’, J
              *Simulium* (*Wilhelmia*) *pseudequinum* Séguy, 1921—A, G, G’, J
              *Simulium* (*Wilhelmia*) *sergenti* Edwards, 1923—G, J

### 3.3. Chorological Information on the Studied Areas and Bibliographic Reports

Below, for each identified species, the geographical coordinates in longitude/latitude are provided, together with the date of collection, locality, province, and altitude in m a.s.l. (metres above sea level), and the number of specimens of each stage of development collected (immature larvae, mature larvae, pupae, and adult).

- *Prosimulium latimucro* (Enderlein, 1925)

Examined material: −3.37468 (longitude)/37.08658 (latitude), 21 May 2015, Sierra Nevada (Monachil district), Granada, 2518 m, three immature larvae, four mature larvae. −3.26496/36.99357, 24 May 2015, Trevélez, Granada, 1454 m, nine immature larvae. −3.03175/37.11666, 26 May 2018, Ferreira, Granada, 2018 m, 19 mature larvae, 98 pupae. −3.27998/36.98380, 25 May 2018, Los Caballeros, Granada, 1466 m, two pupae.

Previous records: Almería [13]; Granada, specimens identified by [14,15] as *P. hirtipes* were considered by [16,17] as *P. latimucro*, [13,16,17,18], specimens identified by [19] as *Prosimulium* sp. by [16,17], specimens identified by [18] as *P. rufipes* were considered as *P. latimucro* by [16,17]; Jaén [13,16].

- *Metacnephia blanci* (Grenier & Theodorides, 1953)

Previous records: Jaén [16].

- *Metacnephia nuragica* (Rivosecchi, Raastad & Contini, 1975)

Previous records: Almería, some specimens recorded as *Cnephia* sp. by [18] later were considered to be *M. nuragica* by [16].

- *Simulium* (*Eusimulium*) *mellah* Giudicelli & Bouzidi, 2000

Previous records: Almería [20].

- *Simulium* (*Eusimulium*) *petricolum* (Rivosecchi, 1963)

Examined material: −3.34783/36.95813, 23 May 2015, Capileira, Granada, 1704 m, 12 mature larvae. −3.41650/38.39656, 27 May 2018, Aldeaquemada, Jaén, 918 m, 12 mature larvae.

Previous records: Almería [13,16,20,21]; Granada, specimens identified as *S. aureum* s.l. by [19] were considered as *S. petricolum* by [16], specimens identified as *S. aureum* by [14] later were considered as *S. petricolum* by [16,17]; Jaén [13,16,22,23].

- *Simulium* (*Eusimulium*) *rubzovianum* (Sherban, 1961)

Examined material: −3.34783/36.95813, 23 May 2015, Capileira, Granada, 1704 m, 17 mature larvae. −3.41650/38.39656, 27 May 2018, Aldeaquemada, Jaén, 918 m, 14 mature larvae.

Previous records: Almería, specimens reported by [16,21] as *Simulium* (*Eusimulium*) *velutinum* (Santos Abreu, 1922) are currently accepted to be *S. rubzvbianum* since according to [24] all the records of *S. velutinum* from mainland Spain must be considered as a synonymy of *S. rubzobianum* [20,25]; Granada, specimens identified as *S. aureum* s.l. by [19] were considered as *S. velutinum* by [6,16] which is currently accepted to be *S. rubzobianum*, the same interpretation is applicable to specimens reported by [16] as *S. velutinum*; Jaén, several specimens identified as *S. aureum* s.l. by [19] later on were considered to be *S. velutinum* by [16,17] which currently must be referred to as *S. rubzovianum* for being a record of mainland Spain, the same occurs with the samples reported by [16] as *S. velutinum*, identical circumstance is applicable to the specimens recorded by [22] as *S. velutinum* whose authors in 2020 reported it as *S. rubzovianum*. In the same manner, the specimens identified by [19] as *S. rubzovianum*? and later considered by [16,17] as *S. velutinum* now must be considered again as *S. rubzovianum* for the reason previously mentioned [23,26].

- *Simulium* (*Nevermannia*) *angustitarse* (Lundström, 1911)

Previous records: Granada [16]; Jaén [16].

- *Simulium* (*Nevermannia*) *ruficorne* Macquart, 1838

Previous records: Almería [16]; Jaén [16,19].

- *Simulium* (*Nevermannia*) *armoricanum* (Doby & David, 1961)

Previous records: Granada, some specimens reported as *Simulium latipes* (Meigen, 1804) by [18] later on were considered to be *S. armoricanum* by [16] and by [13,16,17].

- *Simulium* (*Nevermannia*) *brevidens* (Rubtsov, 1956)

Examined material: −3.41183/37.10407, 21 May 2015, Sierra Nevada (Monachil district), 2099 m, one mature larva.

- *Simulium* (*Nevermannia*) *carthusiense* Grenier & Dorier, 1959

Examined material: −3.37531/37.08665, 21 May 2015, Sierra Nevada (Monachil district), 2506 m, 10 mature larvae, one pupa. −3.37468/37.08658, 21 May 2015, Sierra Nevada (Monachil district), 2518 m, one immature larva, three mature larvae, one pupa. −3.41183/37.10407, 21 May 2015, Sierra Nevada (Monachil district), 2099 m, three pupae.

Previous records: Granada, some specimens identified as *S. latipes* by [18] were considered to be *S. carthusiense* by [16] and by [17], in the same way, specimens identified by [14] as *Simulium* sp. were considered to be *S. carthusiense* by [15] and later reaffirmed by [8,13,16,17,19,27,28].

- *Simulium* (*Nevermannia*) *cryophilum* (Rubtsov, 1959)

Examined material: −3.41259/37.10434, 21 May 2015, Sierra Nevada (Monachil district), 2087 m, 14 mature larvae, one pupa. −3.41183/37.10407, 21 May 2015, Sierra Nevada (Monachil district), 2099 m, four mature larvae. −3.33974/36.96539, 23 May 2015, 1989 m, 21 immature larvae, four pupae. −3.25909/37.00597, 24 May 2015, Trevélez, Granada, 1492 m, six immature larvae, seven mature larvae. −3.27998/36.98380, 25 May 2018, Los Caballeros, Granada, 1466 m, 23 mature larvae, five pupae. −3.28179/36.96487, 25 May 2018, Dúrcal, Granada, 1467 m, two mature larvae, one pupa. −3.03175/37.11666, 26 May 2018, Ferreira, Granada, 2018 m, 53 mature larvae, six pupae. −3.01915/37.07705, 26 May 2018, Bayárcal, Almería, 1713 m, 12 immature larvae, 39 mature larvae, 25 pupae. −3.02040/37.07666, 26 May 2018, Bayárcal, Almería, 1703 m, three immature larvae, six mature larvae.

Previous records: Almería [13,16]; Granada, specimens identified as *S. latipes* by [14] and by [15] were considered to be *S. cryophylum* by [16] and by [13,16,17,28]; Jaén [13,16,22,23].

- *Simulium* (*Nevermannia*) *quasidecolletum* Crosskey, 1988

Examined material: −3.37531/37.08665, 21 May 2015, Sierra Nevada (Monachil district), 2506 m, nine mature larvae. −3.41259/37.10434, 21 May 2015, Sierra Nevada (Monachil district), 2087 m, one mature larva. −3.41183/37.10407, 21 May 2015, Sierra Nevada (Monachil district), 2099 m, one mature larva.

- *Simulium* (*Nevermannia*) *urbanum* Davies, 1966

Authors’ material: −3.03175/37.11666, 26 May 2018, Ferreira, Granada, 2018 m, three pupae.

Previous records: Granada [28].

- *Simulium* (*Nevermannia*) *vernum* Macquart, 1826

Previous records: Almería [13]; Jaén [13].

- *Simulium* (*Simulium*) *bezzi* (Corti, 1914)

Previous records: Granada [13,16,18]; Jaén [16,22,23].

- *Simulium* (*Simulium*) *intermedium* Roubaud, 1906

Examined material: −3.28179/36.96487, 25 May 2018, Dúrcal, Granada, 1467 m, two mature larvae.

Previous records: Almería [6,16]; Granada, some specimens identified as *S. reptans* by [29] were considered to be *S. intermedium* by [16], in like manner some specimens identified as *S. ornatum* by [18] were considered by [16] as *S. intermedium*, [13,16,17,19]; Jaén [13,16,17,19,22,23].

- *Simulium* (*Simulium*) *ornatum* Meigen, 1818

Examined material: −3.33984/36.9654, 23 May 2015, Capileira, Granada, 1987 m, one pupa. −3.28179/36.96487, 25 May 2018, Dúrcal, Granada, 1467 m, two mature larvae. −3.41650/38.39656, 27 May 2018, Aldeaquemada, Jaén, 918 m, one mature larva.

Previous records: Granada [13,16,18,19,30,31]; Jaén [13,16,19,22,23,30,31].

- *Simulium* (*Simulium*) *trifasciatum* Curtis, 1839

Examined material: −3.28179/36.96487, 25 May 2018, Dúrcal, Granada, 1467 m, three mature larvae.

- *Simulium* (*Simulium*) *argyreatum* Meigen, 1838

Previous records: Granada [32]; Jaén [22,23].

- *Simulium* (*Simulium*) sp. aff. *maximum*

Examined material: −3.41259/37.10434, 21 May 2015, Sierra Nevada (Monachil district), 2087 m, two mature larvae, one pupa. −3.41183/37.10407, 21 May 2015, Sierra Nevada (Monachil district), 2099 m, three immature larvae. −3.37531/37.08665, 21 May 2015, Sierra Nevada (Monachil district), 2506 m, one pupa. −3.37468/37.08658, 21 May 2015, Sierra Nevada (Monachil district), 2518 m, one mature larva. −3.26252/37.00456, 24 May 2015, Trevélez, Granada, 1570 m, five immature larvae, two mature larvae.

Previous records: Granada [33]

Note: This taxon was formally not described, and is similar but not identical to the species *Simulium monticola* Friederichs, 1920, and *Simulium maximum* (Knoz, 1963). Old records of these species from Andalusia need to be reviewed [33].

- *Simulium* (*Simulium*) *monticola* Friederichs, 1920

Previous records: Granada, several specimens identified as *S. maximum* (Knoz, 1961) by [13] later were considered to be *S. monticola* by [16] and by [17], in a similar way several specimens identified as *S. variegatum* by [10] subsequently were considered to be *S. monticola* by [14,15,16,18,27,34]; Jaén [16].

- *Simulium* (*Simulium*) *variegatum* Meigen, 1818

Examined material: −3.41164/37.14343, 22 May 2015, Güejar Sierra, Granada, 1087 m, 212 immature larvae, 110 mature larvae, 113 pupae. −3.41486/37.15063, 22 May 2015, Güejar Sierra, Granada, 1051 m, 350 immature larvae, 27 mature larvae, seven pupae. −3.33984/36.96540, 23 May 2015, Capileira, Granada, 1987 m, 13 mature larvae. −3.33974/36.96539, 23 May 2015, Capileira, Granada, 1989 m, one immature larva, four mature larvae. −3.25863/37.00696, 24 May 2015, Trevélez, Granada, 1457 m, 145 immature larvae, 29 mature larvae. −3.27998/36.98380, 25 May 2018, Los Caballeros, Granada, 1466 m, three mature larvae.

Previous records: Almería [16]; Granada, some specimens reported as *S. monticola* and as *S. ornatum* by [18] later were considered as *S. variegatum* by [13,14,16,18,19]; Jaén [16].

- *Simulium* (*Simulium*) *xanthinum* Edwards, 1933

Previous records: Jaén [16,17,22,23,35].

- *Simulium* (*Trichodagmia*) *galloprovinciale* Giudicelli, 1963

Previous records: Jaén [8,16,36].

- *Simulium* (*Wilhelmia*) *equinum* (Linnaeus, 1758)

Examined material: −3.51611/37.16387, 22 May 2015, Pinos Genil, Granada, 779 m, eight immature larvae, 17 mature larvae, 17 pupae.

Previous records: Granada [16,19]; Jaén [16].

- *Simulium* (*Wilhelmia*) *lineatum* (Meigen, 1804)

Examined material: −3.51611/37.16387, 22 May 2015, Pinos Genil, Granada, 779 m, 15 mature larvae.

Previous records: Granada [16,18]; Jaén [16,19].

- *Simulium* (*Wilhelmia*) *pseudequinum* Séguy, 1921

Examined material: −3.51611/37.16387, 22 May 2015, Pinos Genil, Granada, 779 m, three mature larvae.

Previous records: Almería, the specimens identified by [18] as *S. lineatum* were considered as *S. pseudequinum* by [16,19]; Granada, some specimens recorded as *S. lineatum* by [18] afterwards were considered to be *S. pseudequinum* by [16,18,19]; Jaén [16,19,22,23].

- *Simulium* (*Wilhelmia*) *sergenti* Edwards, 1923

Previous records: Granada, some specimens registered as *S. pseudequinum* by [10] later were considered to be *S. sergenti* by [16] and by [16,17]; Jaén [16,19].

Other identifications reported: Granada, the specimens reported as *Simulium* (*Schoenbaueria*) sp. by [18] were considered as *Simulium* (*Rubzovia*) sp. by [16] after ruling out that it is *Simulium* (*Rubzovia*) *lamachi* Doby & David, 1960, because the coloration of the antenna does not match the description of the species. *Simulium* (*Nevermannia*) sp. near *S. cryophilum* [16], specimens reported as *Simulium* (*Byssodon) maculatum* (Meigen, 1804) by [30] and by [31] were considered by [16] as misidentifications since the adult stage was very difficult to identify reliably in Strobl’s time and, as a result, the name *maculatum* (Meigen) was erroneously applied to *S. equinum* and related species of the subgenus *Wilhelmia*; therefore, and according to P. H. Adler, those specimens should be considered as *Simulium* (*Wilhelmia*) spp.; Jaén, specimens recorded as *S. maculatum* by [30] and by [31] were considered by [16] as misidentifications, which should be considered as *Simulium* (*Wilhelmia*) spp., according to P. H. Adler and *Simulium* (*Nevermannia*) sp. [16].

### 3.4. Altitudinal Ranges and Distribution Maps of the Species First Recorded from the Provinces of Granada and Jaén

Among the species recorded from the province of Granada for the first time, *S. quasidecolletum* exhibited a relatively narrow altitudinal range of 419 m, occurring between 2087 and 2506 m. The other two species were recorded only at one collection site—*S. trifasciatum* at 1467 m and *S. brevidens* at 2099 m. An overview of the altitudinal ranges in which the species were found is summarised in Table 3.

**Figure 5:**
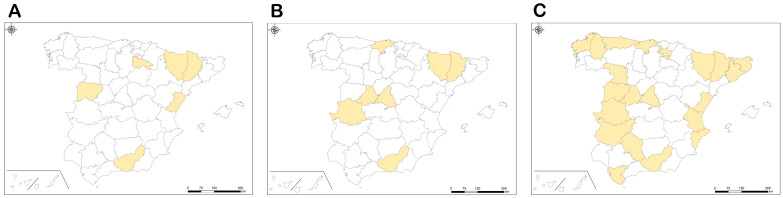
Provincial distribution maps of the species first recorded from the province of Granada: (**A**) *S. brevidens*, (**B**) *S. quasidecolletum,* and (**C**) *S. trifasciatum*.

In relation to the species first recorded from the province of Granada (*S. brevidens*, *S. quasidecolletum*, and *S. trifasciatum*), these records have allowed an increase in their known distribution [37]. As a result, the provincial distribution maps of those species have been updated (Figure 5).

## 4. Discussion

### 4.1. Altitudinal Records

The altitudinal distribution patterns vary greatly among blackfly species. Among the 28 blackfly species recorded from Eastern Andalusia, the narrowest elevation range had *S. ruficorne* in Jaén (50 m), followed by *S. cryophilum* in Almería (97 m), and *S. urbanum* in Granada (182 m). In contrast, other species show exceptionally broader altitudinal distributions. These include *S. petricolum* in Almería, spanning 1557 m; *S. rubzovianum* in Granada, ranging from sea level to 3000 m; *S. intermedium* in Jaén, covering a 1350 m range.

Blackfly species recorded in the present study were found mostly within the previously known altitudinal ranges. Only in the case of *S. lineatum* and *S. equinum* were specimens recorded from higher elevations (779 m), and in the case of *S. urbanum,* were pupae recorded from a lower elevation (2018 m) than previously. The species *S. quasidecolletum*, which was recorded for the first time in Andalusia, had a relatively narrow altitudinal range (419 m). The other two newly recorded species—*S. brevidens* and *S. trifasciatum*—were found only at one collection site.

In the province of Almería, the altitudinal distribution of *S. cryophilum* ranges from 1703 to 1800 m, and it has increased by 97 m [13]. Regarding the altitudinal distributions of the rest of the species reported from the bibliography, four species were reported from one sampling station, such as *S. ruficorne* at <100 m [16], *S. bezzii* at 750 m [16], and *P. latimucro* and *S. vernum*, which were recorded at 1800 m [13]. In addition, seven species were reported from different altitudes. For example, the species with the lowest altitudinal ranges are *S. mellah* with 225 m, which goes from 243 to 468 [20]; *S. variegatum* with 250 m, with a range varying between 500 and 750 m [16]; *M. nuragica* [16,18] and *S. pseudequinum* [20], both of them with 300 m, whose ranges fluctuate between 200 and 500 m. Likewise, two species display an intermediate altitudinal range: *S. intermedium* with 507 m, which goes from 243 [20] to 750 m [16], and *S. rubzovianum* with 650 m, which fluctuates between <100 and 750 m [16]. The species with the widest altitudinal range is *S. petricolum* with 1557 m, which ranges from 243 [20] to 1800 m [13].

With respect to the province of Granada, the elevations at which seven species were collected do not modify their altitudinal ranges. Therefore, the updated altitudinal distribution of *S. carthusiense* fluctuates between 1600 [11,14] and 3000 m [13], although in this study, it has been found between 2099 and 2518 m in Sierra Nevada (Monachil district) (1, 2, 3); in the case of *S. cryophilum*, it varies between 660 [13] and 2750 m [14], since in the present study, it was collected between 1466 and 2099 m in Capileira (3), Dúrcal, Ferreira, Los Caballeros, Sierra Nevada (Monachil district) (2, 4) and Trevélez (3); *S. equinum* ranges from 500 [16] to 800 m [19], since we captured it at 779 m in Pinos Genil; *S. intermedium* from 0 [18] to 2000 m [13] since we sampled it at 1467 m in Dúrcal, *S. ornatum* from 0 to 2500 m [18], even though we collected it between 1467 and 1987 m in Capileira (2) and Dúrcal; *S. variegatum* from 500 [16] to 2200 m [18], though it was found by us between 1051 and 1989 m in Capileira (2, 3), Güejar Sierra (2, 3), Los Caballeros and Trevélez (6). The species with the widest altitudinal range is *S. rubzovianum*, from 0 [16] to 3000 m [19], in spite of the fact that we discovered this species at 1704 m in Capileira (1). However, the elevations at which the other six species were sampled do increase their altitudinal ranges. Secondly, the reports of *P. latimucro* between 1600 [18] and 3050 m [19] have been amplified by 146 m after its capture at 1454 m from a nameless ravine in Trevélez (1), thus the updated altitudinal range varies between 1454 and 3050 m. The species *S. pseudequinum*, reported from 0 to 600 [18], has increased its range by 179 m after its collection at 779 m in the Pinos Genil sampling station from the river de Aguas Blancas, so the current altitudinal range varies between 0 and 779 m. The species *S. urbanum* was reported at 2200 m, and in this study, it was found at 2018 m in the Fereira sampling station in ravine Maja Caco, configuring its altitudinal distribution between 2018 and 2200 m, which means that the altitudinal range includes a variation of 182 m. As for *S. lineatum*, which was reported from <100 to 500 [16], its updated altitudinal range goes from <100 to 779 as a consequence of its collection in Pinos Genil. Eventually, the report of *S. petricolum* between 600 [16] and 1050 m [14] has been increased by 654 m after its discovery at 1704 m in Capileira (1) from ravine de Tejar, so currently it ranges from 600 to 1704 m. The presence of the species *S. argyreatum* has been reported by providing the data regarding the altitude at which the specimens were collected [32]. A part from this, the altitudinal range of the other four species recorded from this province are *S. angustitarse* with 250 m of variation and an altitudinal range that fluctuates between 900 and 1150 m [16], *S. armoricanum* with a oscillation of 400 m and an altitudinal range that goes from 2000 [13] to 2400 m [18], followed by *S. sergenti* with altitude amplitude of 450 m and an altitudinal distribution that ranges from 300 to 750 m [16], and finally *S. bezzii* with 1200 m of variation and an altitudinal distribution that ranges from 300 [18] to 1500 m [13].

With regards to the province of Jaén, the species *S. ornatum*, *S. petricolum* and *S. rubzovianum* collected at 918 m from the stream del Chortal in Aldeaquemada’s sampling station do not change their altitudinal ranges reported in the bibliography. Therefore, they range from 570 [16] to 1600 m [13] in the case of the first species, from 1200 [16] to 1600 m [13] in the second species, and from 200 [19] to 1350 m [16] in the last one. According to the bibliography, from the other 16 species reported from this province, the altitude data of one of them is provided by one of the authors but not by the other; in this way, only the available data is provided for *S. bezzii,* which was collected at 600 m [16], while in [22,23] this information was not provided. With respect to *S. vernum*, this species was only reported from a single sampling site at 1600 m [13]. Subsequently, the altitudinal ranges of the other 14 species are provided ordered from smallest to largest amplitude—the altitudinal distribution of *S. monticola* and *S. ruficorne* covers the smallest width with 50 m, and their altitudinal ranges are 1250–1300 m [16] and 200–250 m [19], respectively: *S. angustitarse*: 580–700 m [16], *S. lineatum*: 480–650 m [16], *S. equinum*: 500–700 m [16], *S. sergenti*: 200–500 m [16,19], *P. latimucro*: 1200–1700 m [13,16], *S. galloprovinciale*: 730–1300 m [16,35], *S. pseudequinum*: 200–800 m [16,19], *S. variegatum*: 600–1300 m [16], *M. blanci*: 480–1300 m [16], *S. xanthinum*: 600–1450 m [16,35], and *S. cryophilum*: 700–1600 m [13,16]. The species with the widest altitudinal range is *S. intermedium*, with 1350 m and an altitudinal distribution that ranges between 250 [19] and 1600 m [13]. In the case of the species *S. argyreatum*, as it happened in the province of Granada, its presence was reported, but again, without providing the data of the altitude at which the specimens were found [17,22], so information in this regard is not supplied.

Table 3 shows the minimum and maximum altitude ranges of the 16 species identified in this study, and the minimum and maximum altitude data for these species from previous studies.

### 4.2. Species Adapted to Cold Waters and High Altitudes

Another notable aspect of the Spanish simuliid fauna is the presence of several species that can be considered relics of the Ice Age, since they are typically stenothermic (adapted to cold water) and are practically isolated in high-altitude mountain refugia such as the Pyrenees, Sierra Nevada or the Central System. That can be the case of *P. latimucro*, *S. cryophilum* or *S.* sp. aff. *Maximum*, among others. These species are primarily concentrated in specific mountain systems that acted as refugia during the post-glacial warming of the Holocene. High-altitude streams still support relict populations of cold-adapted species. For instance, the species *S. cryophilum*, a cold-water specialist often found in fast-flowing mountain streams of high-altitude regions, has been recorded at elevations ranging from 1600 to 2200 m in southern Spain (Jaén, Almería, and Granada; Sierra Nevada) and between 1700 and 2000 m in central and northern mountain systems, including the Central System and the Pyrenees (Madrid, Gerona, Huesca, and Lérida) [6,13,16,33,34]. The species *P. latimucro*, characterised by its preference for cold waters and high altitudes, has also been recorded across major Spanish mountain ranges, including the Sierra Nevada, the Central System, and the Pyrenees, at elevations from 1450 to over 3000 m [6,13,14,16,18,19,26].

### 4.3. Simuliid Species Diversity of This Study Area and Other Regions of Spain

Concerning species diversity in mountain and high mountain aquatic habitats, research carried out over time in different regions of Spain by experts in the study of Diptera of the Simuliidae family allows us to observe certain trends and differences. Although these studies are not directly comparable due to the methodology used, the objective pursued, the number of sampling stations studied, the sampling effort, etc., they nevertheless indicate the approximate species richness in study areas that share the characteristic of having their lotic environments located in high mountain habitats. For example, in the present study, a total of 16 species have been identified in high regions of the province of Granada: four in Jaén, and one in Almería, while in mountainous areas of Catalonia, nine species were reported [13], and in Madrid, 16 species [6], and 15 species in Aragon [13]. Compared to the other mountain or lowland regions of Spain, the biodiversity of blackflies in Eastern Andalusia is high. For example, studies from the Cidacos River Basin [38], Yeguas River [39], Ter River [40], Mijares River [41], and the Serpis River [42] revealed 12–14 species of blackflies. Only in the Júcar River basin were 16 species recorded [43], and in the Pas River basin, 21 species were recorded [44]. Therefore, only studies of extensive areas, such as the Tormes River basin, provide a higher number of species [45].

### 4.4. Haematophagous Species

Of the 16 species identified in the samples taken for this study, six (*S. equinum*, *S. intermedium*, *S. lineatum*, *S. ornatum*, *S. pseudequinum* and *S. variegatum*) stand out for their blood-feeding habits and, therefore, have a significant impact on veterinary medicine, agriculture, tourism and public health due to their annoying bites, the allergic reactions they cause and even diseases caused by the transmission of pathogens such as viruses, protozoa and nematodes. In addition, some of these species have a wide feeding spectrum, while others have a slightly limited range of hosts. For example, some tend to feed on mammalian species, while others tend to obtain their food from the blood of mammals and birds. In the case at hand, these species would be of veterinary, health and economic interest in the areas sampled in the province of Granada, as all of them have been collected in this region. Only the species *S. ornatum* and *S. petricolum* could also be of some concern in the province of Jaén, where they have also been found.

The females of the species *S. argyreatum* and *S. variegatum* bite various farm animals; those of *S. equinum* show a preference for equines as their primary host and may also bite bovids, but display tropism for humans too; females of *S. intermedium* usually bite cattle and horses, as well as humans; *S. lineatum* is biting various mammals and it has also been reported that it sucks human blood [46]; *S. ornatum*, although it prefers bovids, can feed on horses, pigs and humans; and females of *S. pseudequinum* are able to bite cattle, horses and Suidae [47]. In like manner, *S. petricolum* exhibits a preference for avian hosts [24]. In addition to it, in the bibliography consulted, in the area studied, the presence of the species *S. bezzii* was cited, which has been described as an indiscriminate mammalophilic species that can behave as a vector of the myxomatosis virus [48].

Although the extensive livestock farming of cattle, goats, sheep, horses and pigs is in significant decline in Spain, the presence of populations of these species can cause a certain degree of nuisance to both wild and farm animals and domestic animals due to the occasional and seasonal bites they may inflict. Furthermore, as a result of these bites in sensitive areas such as the ears, nostrils, lower abdomen and udders, the repeated accumulation of these bites in search of their necessary intake of blood can condition their feeding, reproduction and production behaviour, causing them nervousness and even affecting their body metabolism. Although it seems that these negative consequences could have a remarkably low or almost imperceptible incidence and impact given the limited distribution of the following species, which in some cases have only been found at one sampling point, as is the case with *S. equinum*, *S. peseudequinum*, *S. intermedium* and *S. lineatum*, this results rather from the distribution of our sampling points, which were concentrated on smaller streams at higher altitudes. In addition, the larger streams and rivers that these species prefer can produce significantly larger numbers of adults, the impact of which can affect a wider area around the breeding sites. Other species could pose a slightly greater threat due to their somewhat wider distribution, as they have been found at three sampling stations in this study, such as *S. ornatum*. From this point of view, the species that could cause the greatest degree of disturbance would be *S. variegatum*, which has been collected at six sampling stations and is the species with the highest population in each of these sampling stations. Finally, the species that could affect avian hosts are *S. petricolum* and *S. rubzovianum*. At all events, lengthy research would be required, using the appropriate methodology to study their bioecology and population patterns throughout the year in order to gain an in-depth understanding of the scope of these blood-feeding species.

## 5. Conclusions

Our findings contribute to a better understanding of the blackfly species diversity in Eastern Andalusia (South-eastern Spain) as well as their altitudinal distribution patterns. This study presents an updated simuliid checklist of the areas of investigation, along with the updated altitudinal range of each species reported from the bibliography and this research. As a result, three species have been recorded for the first time from the province of Granada. A total of 28 species are reported from the three studied provinces, and 16 of them from the present study. The species with the narrowest altitudinal range was *S. urbanum*, followed by *S. equinum* and *S. quasidecolletum*. The widest altitudinal ranges were recorded in *S. variegatum, S. cryophilum and S. ornatum.* Compared to the published data, the altitudinal ranges were updated for species *S. lineatum, S. pseudequinum*, and *S. urbanum*. Future taxonomical work on the studied species using an integrated morphological, molecular and cytotaxonomic approach is expected, with the aim of verifying the morphological identifications as well as the presence of possible cryptic species.

## Figures and Tables

**Table 1:** Data on the studied sampling stations of blackflies from the provinces of Almería, Granada, and Jaén, Autonomous Region of Andalusia, South-eastern Spain.

Province	Locality	River	Longitude (W-E)	Latitude (N-S)	Altitude (m)	Date
Almería	Bayárcal (1)	Ravine/Barranco del Hielo	−3.01915	37.07705	1713	26 May2018
	Bayárcal (2)	Nameless ravine (of Stream/Arroyo del Palancón)	−3.02040	37.07666	1703	26 May 2018
Granada	Capileira (1)	Ravine/Barranco del Tejar	−3.34783	36.95813	1704	23 May 2015
	Capileira (2)	Baja irrigation ditch/Acequia Baja	−3.33984	36.96540	1987	23 May 2015
	Capileira (3)	Baja irrigation ditch/Acequia Baja	−3.33974	36.96539	1989	23 May 2015
	Dúrcal	Ravine/Barranco de los Alisos	−3.28179	36.96487	1467	25 May 2018
	Ferreira	Ravine/Barranco Maja Caco	−3.03175	37.11666	2018	26 May 2018
	Güejar Sierra (1)	River/Río Maitena	−3.41486	37.15063	1051	22 May 2015
	Güejar Sierra (2)	River/Río Genil	−3.41164	37.14343	1087	22 May 2015
	Güejar Sierra (3)	River/Río Maitena	−3.41486	37.15063	1051	22 May 2015
	Los Caballeros	Ravine/Barranco de la Bina	−3.27998	36.98380	1466	25 May 2018
	Pinos Genil	River/Río de Aguas Blancas	−3.51611	37.16387	779	22 May 2015
	Sierra Nevada (Monachil district) (1)	Stream/Arroyo de San Juan	−3.37468	37.08658	2518	21 May 15
	Sierra Nevada (Monachil district) (2)	Nameless ravine (of River/Río Monachil)	−3.41183	37.10407	2099	21 May 2015
	Sierra Nevada (Monachil district) (3)	Stream/Arroyo de San Juan	−3.37531	37.08665	2506	21 May 2015
	Sierra Nevada (Monachil district) (4)	Nameless ravine (of River/Río Monachil)	−3.41259	37.10434	2087	21 May 2015
	Sierra Nevada (Monachil district) (5)	Stream/Arroyo de San Juan	−3.37531	37.08665	2506	21 May 2015
	Trevélez (1)	Nameless ravine (of River/Río Trevélez)	−3.26496	36.99357	1454	24 May 2015
	Trevélez (2)	River/Río Trevélez	−3.26211	37.0004	1457	25 May 2018
	Trevélez (3)	Ravine/Barranco las Majadillas	−3.25909	37.00597	1492	24 May 2015
	Trevélez (4)	Ravine/Barranco Porras	−3.26252	37.00456	1570	24 May 2015
	Trevélez (5)	River/Río Chico	−3.27034	37.00223	1547	23 May 2015
	Trevélez (6)	River/Río Trevélez	−3.25863	37.00696	1490	24 May 2015
Jaén	Aldeaquemada	Stream/Arroyo del Chortal	−3.41650	38.39656	918	27 May 2018

**Table 2:** Specimens of blackflies (A—Almería, G—Granada, and J—Jaén).

Species	No. of Immature Larvae	No. of Mature Larvae	No. of Pupae	Province
*Prosimulium latimucro* s.l.	12	23	100	G
*Prosimulium* sp.	3	0	0	G
*Simulium* (*Eusimulium*) *petricolum*	0	24	0	G, J
*S.* (*E.*) *rubzovianum*	0	31	0	G, J
*Simulium* (*Eusimulium*) sp.	0	0	15	G, J
*S.* (*Nevermannia*) *brevidens*	0	1	0	G
*S.* (*N.*) *carthusiense*	1	13	5	G
*S.* (*N.*) *cryophilum* s.l.	42	148	42	A, G
*S.* (*N.*) *quasidecolletum*	0	11	0	G
*S.* (*N.*) *urbanum*	0	0	3	G
*Simulium* (*Nevermannia*) sp.	1	0	0	G
*S.* (*Simulium*) *intermedium*	0	2	0	G, J
*S. (S.*) *ornatum* s.l.	0	3	1	G, J
*S. (S.*) *trifasciatum*	0	3	1	G
*Simulium* (*Simulium*) sp. aff. *maximum*	8	5	2	G
*S. (S.*) *variegatum*	708	186	120	G
*S.* (*Wilhelmia*) *equinum* s.l.	8	17	17	G
*S.* (*W.*) *lineatum*	0	15	0	G
*S.* (*W.*) *pseudequinum* s.l.	0	3	0	G
*Simulium* sp.	1134	1	0	A, G, J
Total	1917	508	306	

**Table 3:** Altitudinal ranges of blackfly species recorded in Eastern Andalusia. New values that contribute to increasing the altitudinal ranges of these species from previously published data are written in bold. The numbers in brackets indicate the bibliographic references for the data contained in Table 3.

Species	Altitude Range (m)			
	Published Data		Present Study	
	Min	Max	Min	Max
*Prosimulium latimucro*	1200 [16]	3050 [16,17,19]	1454	2518
*Simulium petricolum*	243 [20]	1800 [13,16]	918	1704
*S. rubzovianum*	0 [16]	3000 [16,17,19]	918	1706
*S. brevidens*			**2099**	**2099**
*S. carthusiense*	1600 [16,19]	3000 [8,13,16]	2099	2518
*S. cryophilum*	660 [13,16]	2750 [14,16,17]	1466	2099
*S. quasidecolletum*			**2087**	**2506**
*S. urbanum*	2200 [28]	2200 [28]	**2018**	2018
*S. intermedium*	0 [16,18]	2000 [13,16,17]	1467	1467
*S. ornatum*	0 [16,18]	2500 [16,18]	918	1987
*S. trifasciatum*			**1467**	**1467**
*S.* sp. aff. *maximum*	2060 [33]	2080 [33]	**1570**	**2518**
*S. variegatum*	500 [16]	2200 [16,18]	1051	1989
*S. equinum*	500 [16]	800 [16,19]	779	779
*S. lineatum*	<100 [16]	650 [16]	779	**779**
*S. pseudequinum*	0 [16,18]	800 [16]	779	779

## Data Availability

The original contributions presented in this study are included in the article and Supplementary Material. Further inquiries can be directed to the corresponding author.

## Supplementary Materials

The following supporting information can be downloaded at: https://www.mdpi.com/article/10.3390/insects17030267/s1, Figure S1: Geographical positioning of the provinces of Almería and Granada within Spain (left side). Location of the lotic water bodies prospected along with the sampling stations (right side). Detailed depiction of the water flows, and each sampling station of the province of Almería (below). The numbers correspond to the sample codes, which can be found in the accompanying tables; Figure S2: Geographical positioning of the province of Granada within Spain (left side). Location of the lotic water bodies prospected along with the sampling stations within the black circle (right side). Detailed depiction of the water flows, and each sampling station located inside the black circle (below). The numbers correspond to the sample codes, which can be found in the accompanying tables; Figure S3: Geographical positioning of the province of Granada within Spain (left side). Location of the lotic water bodies prospected along with the sampling stations within the black circle (right side). Detailed depiction of the water flows, and each sampling station located inside the black circle (below). The numbers correspond to the sample codes, which can be found in the accompanying tables; Figure S4: Geographical positioning of the province of Jaén within Spain (left side). Location of the lotic water body prospected along with the sampling station (right side). Detailed depiction of the water flow, and the sampling station (below).

## Abbreviations

The following abbreviations are used in this manuscript:

*E.*	*Eusimulium*
*N.*	*Nevermannia*
*S.*	*Simulium*
*W.*	*Wilhelmia*
m	metres
sp.	species
aff.	affinity
a.s.l.	above sea level
s.l.	sensu lato
A	Almería
G	Granada
J	Jaén
