# Supplementary material for: Simuliids (Diptera: Simuliidae) from Eastern Andalusia (Spain): Update and New Contributions
Source: Insects. 2026 Mar 2;17(3):267. doi: 10.3390/insects17030267 (PMC13026254; doi:10.3390/insects17030267)
Supplement: Supplementary file 1 [file insects-17-00267-s001.zip › insects-4139749-supplementary.pdf]

Supplementary Materials

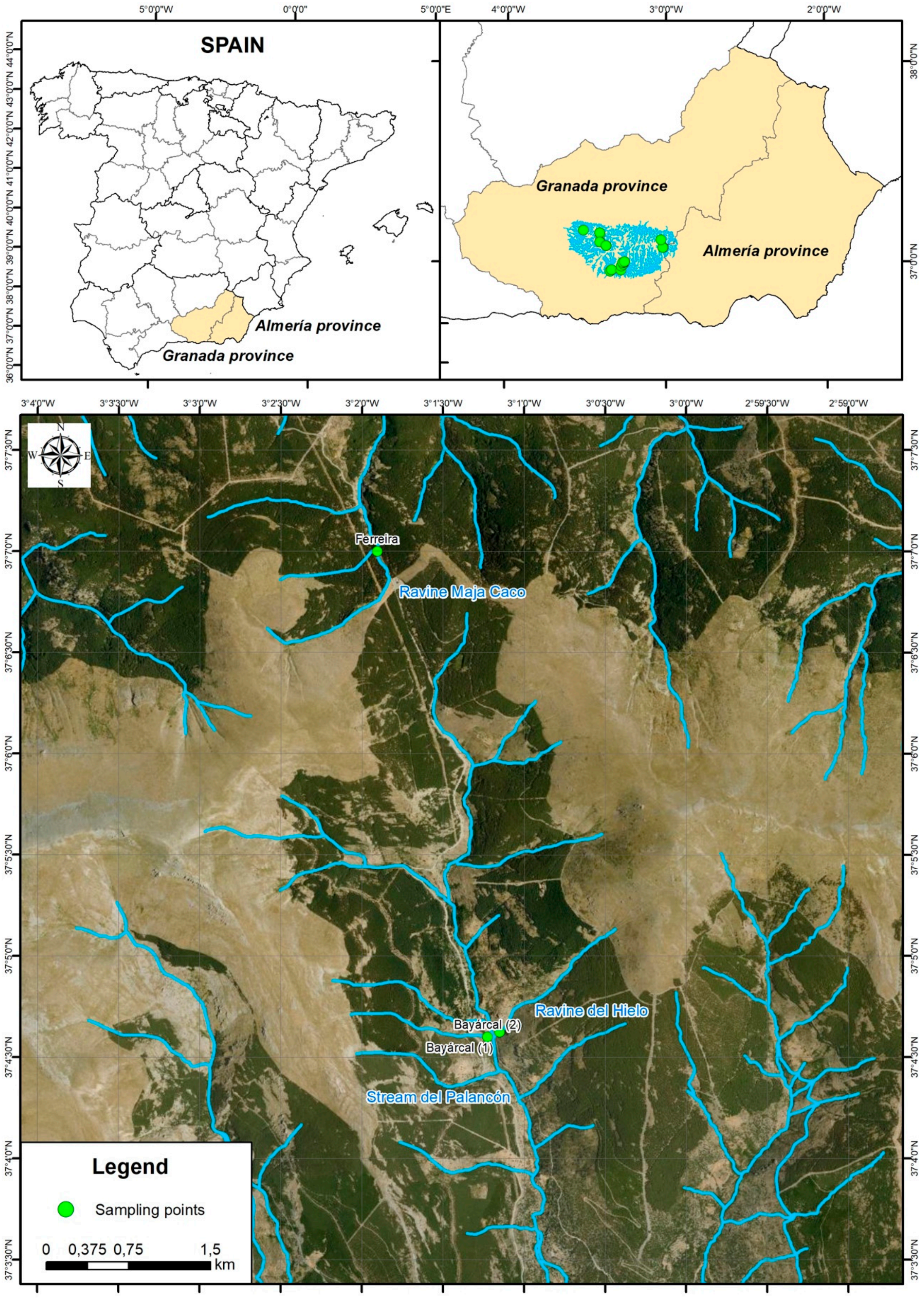

Figure S1. Geographical positioning of the provinces of Almería and Granada within Spain (left side). Location of the lotic water bodies prospected along with the sampling stations (right side). Detailed depiction of the water flows, and each sampling station of the province of Almería (below). The numbers correspond to the sample codes, which can be found in the accompanying tables.

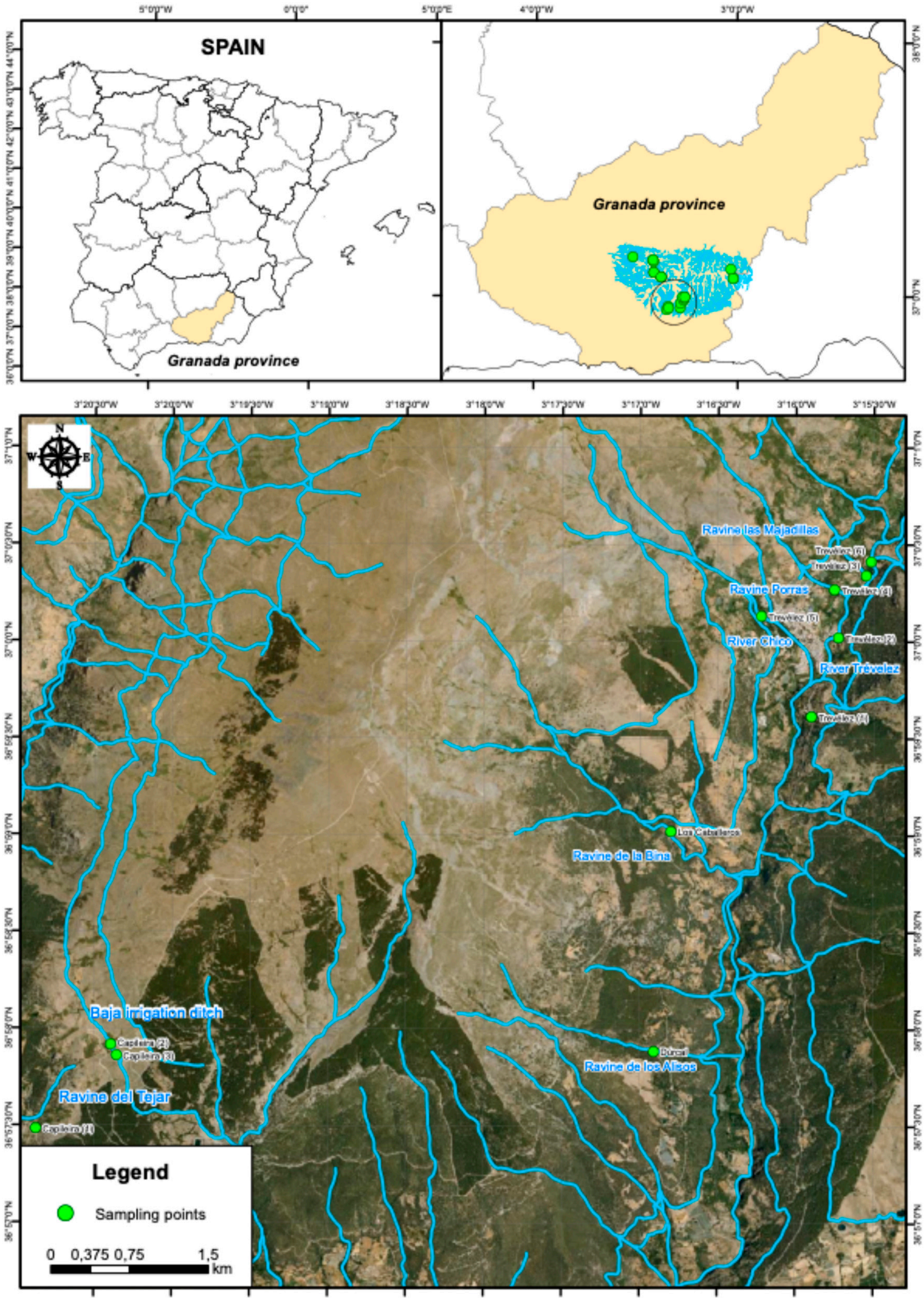

Figure S2. Geographical positioning of the province of Granada within Spain (left side). Location of the lotic water bodies prospected along with the sampling stations within the black circle (right side). Detailed depiction of the water flows, and each sampling station located inside the black circle (below). The numbers correspond to the sample codes, which can be found in the accompanying tables.

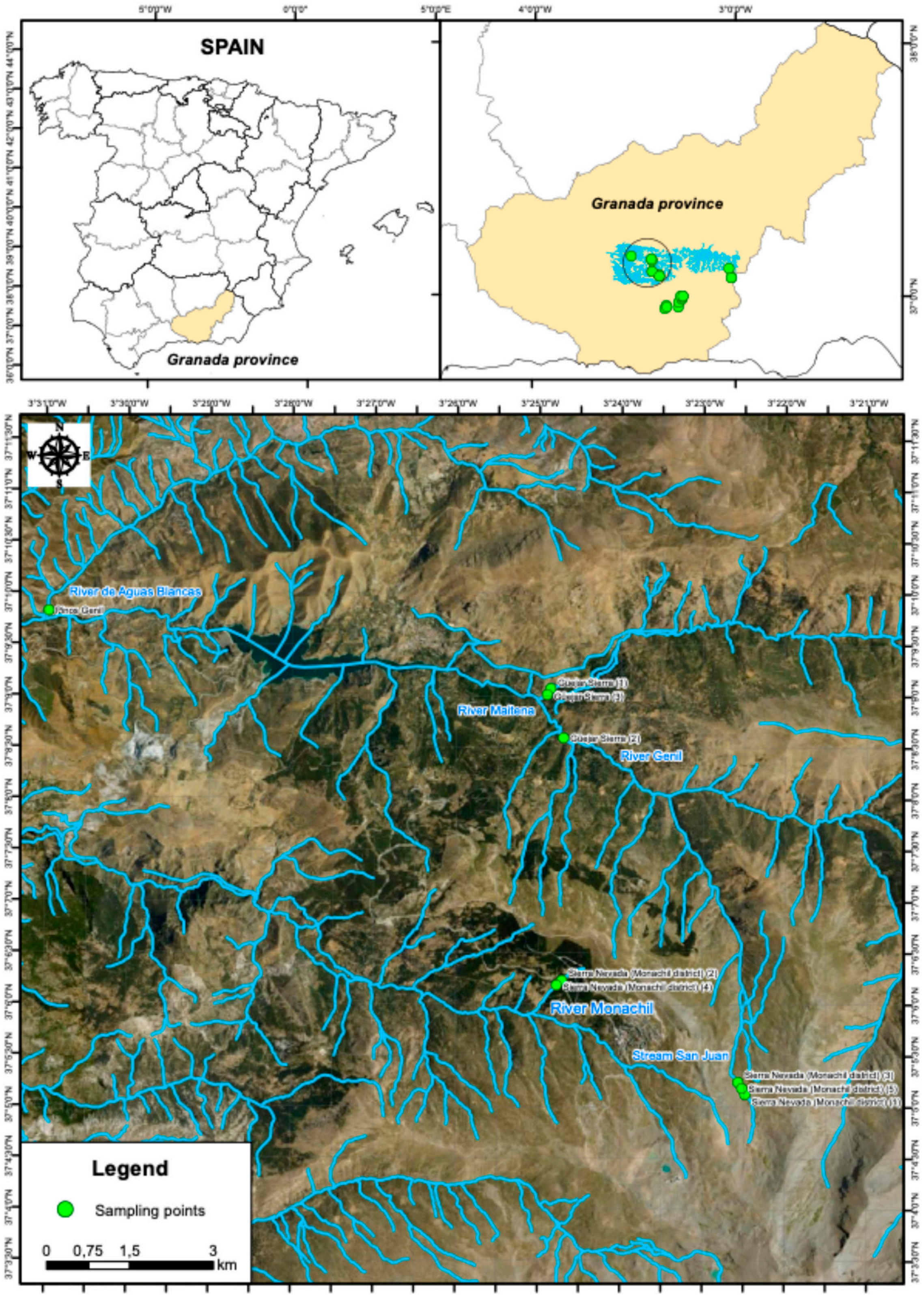

Figure S3. Geographical positioning of the province of Granada within Spain (left side). Location of the lotic water bodies prospected along with the sampling stations within the black circle (right side). Detailed depiction of the water flows, and each sampling station located inside the black circle (below). The numbers correspond to the sample codes, which can be found in the accompanying tables.

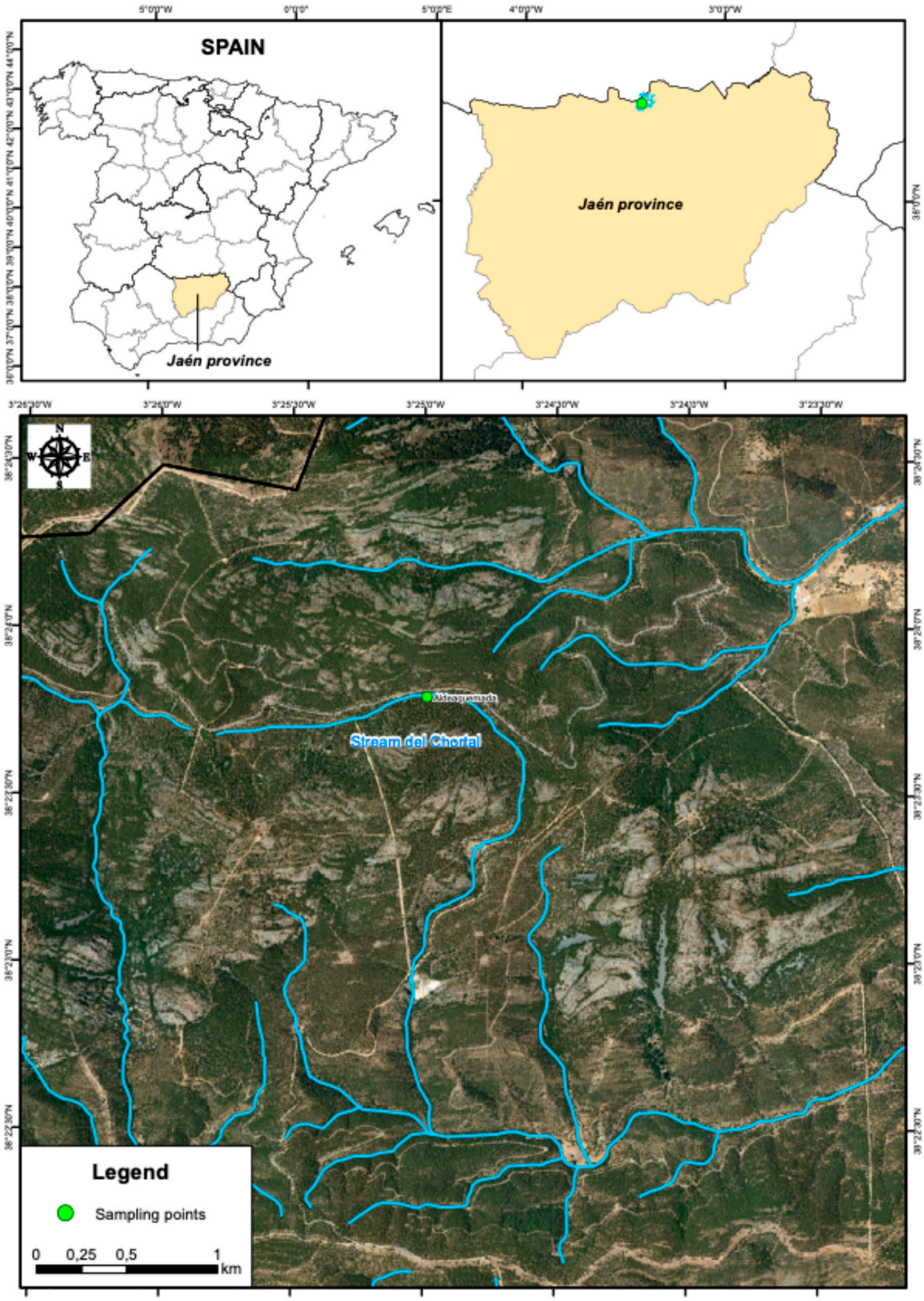

Figure S4. Geographical positioning of the province of Jaén within Spain (left side). Location of the lotic water body prospected along with the sampling station (right side). Detailed depiction of the water flow, and the sampling station (below).
